# Spatiotemporal variability and environmental factors of harmful algal blooms (HABs) over western Lake Erie

**DOI:** 10.1371/journal.pone.0179622

**Published:** 2017-06-28

**Authors:** Di Tian, Gengxin Xie, Jing Tian, Kuo-Hsin Tseng, C. K. Shum, Jiyoung Lee, Song Liang

**Affiliations:** 1 Department of Crop, Soil, and Environmental Sciences, College of Agriculture, and Climate, Human and Earth System Sciences Cluster, Auburn University, Auburn, Alabama, United States of America; 2 College of Urban Construction and Environmental Engineering, Chongqing University, Chongqing, China; 3 Department of Biostatistics, College of Public Health and Health Professions, University of Florida, Gainesville, Florida, United States of America; 4 Center for Space and Remote Sensing Research, National Central University, Taoyuan, Taiwan; 5 School of Earth Sciences, The Ohio State University, Columbus, Ohio, United States of America; 6 Division of Environmental Health Sciences, College of Public Health, The Ohio State University, Columbus, Ohio, United States of America; 7 Department of Environmental and Global Health, College of Public Health and Health Professions, and Emerging Pathogens Institute, University of Florida, Gainesville, Florida, United States of America; Koninklijk Nederlands Instituut voor Onderzoek der Zee, NETHERLANDS

## Abstract

Over the past decades, numerous studies have been carried out in understanding causes of Harmful Algal Blooms (HABs) and their dynamics, yielding great knowledge in this field. Lake Erie, the fourth-largest lake of the five Great Lake, is among those highly vulnerable to the impacts of HABs and has received substantial attention from the public, water management sectors, and academic field. Building upon previous work, this study aims to characterize spatiotemporal variability of Chlorophyll a (Chl-a), which is an important indicator of HABs, and to explore relative importance of environmental factors associated with HABs in the west Lake Erie. Ten years of biweekly Chl-a information over western Lake Erie were derived from MERIS data at the pixel scale. Based on the MERIS-derived information high concentrations of Chl-a were observed in the south near shore area in spring and fall and in the west corner area of western Lake Erie in all three seasons except winter. Wavelet analysis suggested that the 0.5- and 1-year periods were dominant modes for the Chl-a series. The Multivariate Adaptive Regression Splines (MARS) analysis was performed to explore factors associated with the dynamics of Chl-a. The results suggested that overall both phenological (e.g. wind) and ecological (e.g. nutrient levels) factors exhibited significant correlations with the remotely-sensed imagery based observations of Chl-a despite spatial and temporal variations. The important phenological and ecological factors include solar radiation and wind speed in spring, water temperature, solar radiation, and total Kjeldahl nitrogen concentration in summer, wind speed in fall, and water temperature and streamflow in winter. Both consistency and differences of findings of the study with others in the region may suggest strengths and limitations of the remotely sensed imagery-based analysis, offering valuable information for future work.

## Introduction

Over the past few decades, the issues and complexity of determinants of harmful algal blooms (HABs) have expanded in their scopes, raising substantial global concerns over their environmental and public health impacts [1–5]. Although more than 300 algal species may cause the blooms, only one-third of them are deemed toxic algae [1, 6]. Worldwide, many terrestrial water environments are under emerging, ongoing, and increasing impacts of HABs [6–8] due to a wide array of factors ranging from eutrophication of anthropologic origins to environmental change such as global warming [2, 6–7, 9–11]. Cyanobacteria in both freshwater and coastal marine environments, is among those of particular concern, posing great environmental and health impacts [2, 10, 12–15].

The Great Lakes, consisting of a series of interconnected freshwater lakes, hold about 1/5 of the world’s and about 95% of the US freshwater supply and are the world’s largest and biologically diverse freshwater resource [16]. Lake Erie, the fourth-largest, the shallowest, smallest in water volume, and most southern among the Great Lakes, is surrounded by somewhat more urbanized environment and extensive agricultural land-use than any other lake regions; as such, Lake Erie, in particular its western basin, is subject to substantial impacts from excessive nutrient loadings [17]. In addition, due to its geographical characteristics, Lake Erie is also vulnerable to extreme weather events [18, 19]. Over the past decades, western Lake Erie has witnessed increasingly intense algal blooms including cyanobacterial blooms [20–24], with a record-setting algal bloom event occurred in 2011 [25]. Many studies have been carried out aiming to characterize temporal variability of cyanobacterial blooms or hypoxic extent, and reveal causes that may be associated with the blooms, thereby informing possible mitigation strategies and early warning, and predicting future trends [23, 25, 26–30]. For example, Stump et al. [23] conducted correlation analysis between bloom area and environmental factors and found that the total spring discharge or the spring total phosphorus load appeared sufficient to predict the bloom magnitude over western Lake Erie; Michalak et al. [25] analyzed long-term trends of different factors (e.g. nutrient loading and meteorological conditions) and concluded that the record-breaking 2011 bloom in Lake Erie was largely driven by the trends of those factors; Obenour et al. [29] developed a Bayesian hierarchical model to include uncertainty in forecasting the bloom size in Lake Erie; Zhou et al. [30] analyzed bloom extent in Lake Erie and developed a model for explaining their interannual variability. Most of those studies focused on the spatial extent or a short period of the HABs indices over the western Lake Erie. Improved knowledge of spatial heterogeneities and long-term temporal variability might help to gain mechanistic understanding on the dynamics of HABs movements or distributions. For example, it is well known that nutrient loadings are a driving factor of the occurrence of HABs, do some well-known phenological factors such as wind, play a role in the distribution of HABs? If so, how do they interact with other ecological factors, such as nutrient loading and temperature, influence growth and death, then distribution of HABs? Chlorophyll a (Chl-a) is one of the most important indicators of HABs, which can be derived from remotely sensed imagery at high spatial-temporal resolution (pixel scale) with full coverages over Lake Erie. Using remote sensing derived information, this study investigated spatiotemporal heterogeneities of Chl-a in western Lake Erie and explored the relative importance of the environmental factors of varying natures in correlating with the distribution of Chl-a based on the remote sensing information.

## Materials and methods

### Indicators of HABs

MERIS is the remote sensing payload onboard Envisat aimed to monitor the land, ocean, and atmosphere change in solar reflective spectral range. MERIS had been operated between May 2002 and April 2012 during Envisat’s mission lifetime together with various sensors such as radar altimetry and synthetic aperture radar. The spectral range of MERIS covers visible to near infrared, including 15 bands across 390–1040 nm with bandwidth programmable between 2.5–30 nm. Concentrations of Chl-a, the indicator of HABs Chlorophyll a (Chl-a) (Table 1) were estimated based on an approach developed by Simis et al. [31–32]. Detailed procedures were also detailed in our previous work [33] and a description of this approach is described as follows.

**Table 1:** Data summary.

	Symbol	Unit	Source
**Indicator of HABs**			
Chlorophyll-a	Chl-a	μg/L	MERIS
**Environmental factors** (ecological and phenological)			
Total Phosphorus	TP	μg/L	NCWQR
Soluble Reactive Phosphorus	SRP	μg/L	NCWQR
Nitrate plus nitrite concentration	NO23	μg/L	NCWQR
Total Kjeldahl Nitrogen concentration	TKN	μg/L	NCWQR
Discharge	Q	cfs	NCWQR
Precipitation	PCP	mm	NOAA CPC
Wind speed	Wind	m/s	NDBC (No. SBIO1 and 45005)
Water temperature	WTMP	F	NDBC (No. 45005)
Solar radiation	Rs	W/m^2^	NASA

According to Simis et al. [32], band 7 is centered at 665 nm and supports the estimation of Chl-a at an independent spectrum. The estimate of Chl-a via wavelength near 665 nm is described as [31]:

$$\alpha_{chl}\left(665\right)=\left\{\left[\left(\frac{B\left(705\right)}{B\left(665\right)}\right)\times \left(a_{w}\left(709\right)+b_{b}\right)\right]-b_{b}-a_{w}\left(665\right)\right\}\times \gamma^{-1}$$ (1)

where

*B*(665) = Band 7 water-leaving reflectance centered at 665 nm for measuring a peaked shoulder in Chl-a absorbance spectra
*a*_*w*_(665) = pure water absorption at 665 nm
*γ* = 0.68, estimated Chl-a absorption

The Chl-a concentrations derived from MERIS were extracted over the western Lake Erie (82.6^o^ to 83.5^o^W and 41.5^o^ to 42.1^o^N). The Chl-a concentrations derived from MERIS had a spatial resolution of approximately 300 meters and were interpolated into 1000 meters to avoid data voids. The original revisit period of MERIS swath scanning is 2–3 days at this location, however, because of the noise level and the number of missing values caused by cloudiness in the imagery, the time scale of this study has been downscaled to biweekly. For instance, in order to get a high-quality time series of Chl-a, 14-day composites of Chl-a from 5 January 2002 to 21 March 2012 were calculated by averaging Chl-a at each pixel available from any of the daily images within a given 14-day time period. All the available images for each 14 days were taken into account for temporal binning. The pixels with more than 50% missing values in the converted 14-day time series were removed, which were mostly near the boundary of water due to complex atmosphere/radiance correction and seasonal changes of lake water levels.

A two-step procedure was used to fill the missing data points of the Chl-a derived from MERIS. In the first step, the k-nearest neighbor (KNN) imputation [34] was employed to fill missing values spatially (i.e., in each pixel with less than 50% of missing values). The KNN imputation filled the missing values in any pixel with the weighted average of the corresponding values from its k nearest-neighbor pixels. In this study, the nearest-neighbor time series were the closest time series in the Euclidean distance; the weights were inversely proportional to the distances from the neighboring time series; k was determined by the square root of the pixel length [35]. However, the KNN imputation was not able to fill these time gaps when the data are missing simultaneously. In the second step, after the KNN imputation we directly filled some small data gaps (approximately 20% of the Chl-a data) using the mean of the available data within the ±14-day window of its neighbor years.

### Environmental factors

Nine environmental (grouped as ecological and phenological) variables associated with nutrient loading and meteorological measurements were collected from different sources (Table 1) and were included in the analyses detailed below.

Nutrient loading variables including total phosphorus (TP), soluble reactive phosphorus (SRP), nitrate plus nitrite concentration (NO23), total Kjeldahl nitrogen concentration (TKN), and discharge (Q) were retrieved from the dataset by the National Center for Water Quality Research at Heidelberg University (NCWQR) which maintains water quality monitoring for Maumee River, Raisin River, and Sandusky River. Watersheds of those three rivers are dense in population and contribute major nutrient loadings to western Lake Erie. The NCWQR dataset includes sub-daily (e.g., hourly) observations and they were converted to daily time series by averaging multiple measurements within a day. The daily time series from 5 January 2002 to 21 March 2012 for each of the three monitoring sites were collapsed to bi-weekly series. The single biweekly time series of nutrient loading and discharge were averaged over the three monitoring sites.

Gridded 0.25^o^x0.25^o^ daily precipitation (PCP) data covering 5 January 2002 to 21 March 2012 over western Lake Erie (82.6^o^ to 83.5^o^W and 41.5^o^ to 42.1^o^N) were extracted from the National Oceanic and Atmospheric Administration (NOAA) Climate Prediction Center (CPC) United States Unified Precipitation data (http://www.esrl.noaa.gov/psd/data/gridded/data.unified.html). The biweekly precipitation series were converted from the spatial average of daily precipitation series over western Lake Erie. Hourly surface wind speed (Wind) and water temperature (WTMP) data were retrieved from the NOAA National Data Buoy Center (NDBC) website for Stations 45005 (http://www.ndbc.noaa.gov/station_page.php?station=45005) and SBIO1 (http://www.ndbc.noaa.gov/station_page.php?station=sbio1) and were converted into daily values. These two buoy stations were selected since they had a relatively complete Wind and WTMP data record. We assume that Wind and WTMP at the two stations were representative of the conditions over the study area. The biweekly surface wind speed and water temperature series were then converted from the daily values and were expressed as the average of the two time series from Stations 45005 and SBIO1. Surface wind speed did not have missing values. Missing values of the biweekly water temperature series (approximately 30% of the entire series) were filled using means of the available data within the ±14-day window of its neighbor years, the same method was used for the Chl-a missing data. Gridded 0.125^o^x0.125^o^ hourly solar radiation (Rs) data from 5 January 2002 to 21 March 2012 over western Lake Erie (82.6^o^ to 83.5^o^W and 41.5^o^ to 42.1^o^N) were extracted from the forcing data for Phase 2 of the North American Land Data Assimilation System (NLDAS-2) (http://disc.sci.gsfc.nasa.gov/hydrology/data-holdings*)* and were converted into daily series. The biweekly solar radiation series were the spatial average of daily solar radiation over western Lake Erie.

### Wavelet analysis

To explore temporal pattern (e.g. periodicity) of Chl-a, continuous wavelet transformation (CWT) was used to decompose a time series from the time domain to time-frequency (or time-period) domain and detect localized intermittent periods of a time series. A detailed description of CWT is provided by Torrence and Compo [36] and here is a brief summary of CWT relevant to this study. The CWT of a discrete sequence *x*_*n*_ is defined as the convolution of *x*_*n*_ with a scaled and translated version of *ψ*_*o*_*(h)*:

$$W_{n}\left(s\right)=\sum_{n'=0}^{N-1}x_{n'}\psi^{*}\left[\frac{\left(n'-n\right)\delta t}{s}\right]$$ (2)

where *n* is the localized time index, *s* is the scale, *δt* is the sampling period, *N* is the number of points in the time series and the (*) indicates the complex conjugate. The wavelet power spectrum is defined as |*W*_*n*_(*s*)|^2^. The large wavelet power indicates dominant period *ψ*_*o*_*(h)* and is the wavelet function (or mother wavelet). Morlet wavelet function is used in this research and is written as:

$$\psi_{0}\left(\eta \right)=\pi^{-1/4}e^{i\omega_{0}\eta }e^{-\eta^{2}/2}$$ (3)

where *η* is the nondimensional time factor, *ω*_o_ is the nondimensional frequency. Global wavelet spectrum is calculated as:

$${\bar{W}}^{2}\left(s\right)=\frac{1}{N}{\sum_{n=0}^{N-1}\left|W_{n}\left(s\right)\right|}^{2}$$ (4)

The Cone of Influence is the region of the wavelet spectrum in which edge effects cannot be ignored [36]. The statistical significance (95%) of the wavelet power spectrum was assessed relative to the null hypothesis in that the time series is generated as red noise.

### Multivariate Adaptive Regression Splines (MARS)

The relationships between environmental variables (both ecological and phenological) and remote sensing based observations of Chl-a concentrations might be are complex (e.g. additive and interactions of environmental variables of distinct natures), here we used a non-parametric approach, termed Multivariate Adaptive Regression Splines (MARS), to explore the relative importance of the environmental variables. MARS is a well-established machine learning method and a valuable analytic tool that provide more power and flexibility (e.g. relaxing assumption) to model the relationships that involve interactions, which has been widely applied in many fields including environmental science and public health [37–39].

The principle of MARS is to partition the predictor variable space into disjoint regions based on the knot and fit piecewise spline (basis function) to each region [40]. The final model is the combination of those basis functions. A general MARS model can be written as:

$$Y=\sum_{i=1}^{k}\beta_{i}B_{i}(X)+\varepsilon$$ (5)

where Y is the response variable (Chl-a in this study); X is the vector of predictors, including the nine environmental variables; each *β*_*i*_ is a constant coefficient, which can be estimated by minimizing sum of squared residuals ε; *k* is the number of basis function included in the final model; *B*_*i*_ is the *i*th basis function that represents one of the three forms, a constant 1, a hinge function, or a product of two or more hinge functions. The hinge function takes the form:

$${\left(x-t\right)}_{+}=\{\begin{array}{c} x-t,\;if\;x>t,\\ 0,\;otherwise, \end{array}\;and\;{\left(t-x\right)}_{+}=\{\begin{array}{c} t-x,\;if\;x<t,\\ 0,\;otherwise. \end{array}$$ (6)

where *x* is a predictor variable; *t* is constant, called the knot; the “+” means the positive part. More than one pair of basis functions (or more than one knot) can be specified for a predictor variable, allowing complex non-linear relationships to be fitted.

A detailed description of the MARS model building can be found in Hastie et al. [41]. A brief introduction is given as follows. MARS model building includes two basic parts, the forward and the backward pass. The forward pass is a so-called greedy fitting algorithm. It starts with a model with only intercept term and then adds basis functions in pairs to the model that gives the maximum reduction in the sum of squared residuals. This process of adding terms continues until the max number of terms in the model is reached or the change in residual error becomes negligible. The maximum number of basis functions can be pre-specified by the user. The model built by the forward pass is usually overfitted and the backward pass prunes the model and finds the optimal model. It deletes the least effective term one at a time from the model until it finds the best model. The models are compared using the Generalized Cross Validation (GCV) criterion, which trades off goodness-of-fit against model complexity. The models with lower values of GCV are better. The GCV criterion is given by:

$$GCV(\lambda )=\frac{\sum_{i=1}^{N}{\left(y_{i}-{\hat{f}}_{\lambda }(x_{i})\right)}^{2}}{{\left(1-M(\lambda )/N\right)}^{2}}$$ (7)

where $\left(y_{i}-{\hat{f}}_{\lambda }(x_{i})\right)$ is the difference between the observed and the model estimate; N is the number of observations; *M(λ)* is the effective number of parameters of the model, which accounts for both the number of basis functions and the number of knots. The readers can refer to Hastie et al. (2009) for the detailed calculation of *M(λ)*.

It is noted that the environmental variables are likely to be highly correlated, causing so-called multicollinearity. It would be less desirable to implement MARS approach in choosing among predictors when multicollinearity is present [42]. In an attempt to identify multicollinearity, we propose to use the Variance Inflation Factor (VIF) statistic to screen for multicollinear variables [43]. In VIF, each predictor is regressed against other predictors. The VIF is calculated based on the coefficient of determination (*R*^*2*^) of the regression model:

$$VIF_{j}=\frac{1}{1-R_{j}{}^{2}}$$ (8)

where the VIF for variable *j* is the reciprocal of the inverse of *R*^*2*^ from the regression. VIF is calculated for each variable. A variable is usually considered to be subject to severe multicollinearity if its VIF is greater than 10 [44] and is sequentially removed from the analysis. In our study, the VIF analysis was conducted for each season (Spring: March to May, Summer: June to August; Fall: September to November; Winter: December to February).

In this study, the VIF selected environmental variables averaged over western Lake Erie were used as predictors for Chl-a in the MARS models for each season and each pixel over the western Lake Erie, which can be represented as *y*_*i*,*j*_ = *f*(*x*) + *ε*, where *y* is the Chl-a concentrations at pixel *i* and season *j*, *x* represents environmental variables for the entire western Lake Erie. We tested different lagged relationships between predictors and Chl-a using linear regression. It was found that the predictors in the same season yielded the best result. Therefore, predictors in the same season with Chl-a were used in MARS models without considering lagged relationship. The R earth package (version 3.2–6) (http://CRAN.R-project.org/package=earth) was used to fit MARS models and to quantify environmental variables in terms of their importance. In the R Earth package, three criteria were used to assess variable importance in a MARS model [45]. The first criterion was to count the number of model subsets (generated by the pruning pass) that include the variable. Variables that are included in more subsets are considered more important. The second criterion was to calculate the reduction in the residual sum of squares for each subset relative to the previous subset. The variables yielding larger net reductions in the residual sum of squares are considered more important. The third criterion was the same as the second criterion except that it uses GCV instead of the residual sum of squares to evaluate the effect of adding or deleting variables on the model.

## Results and discussion

### Spatial characteristics of Chl-a in different seasons

**Fig 1:**
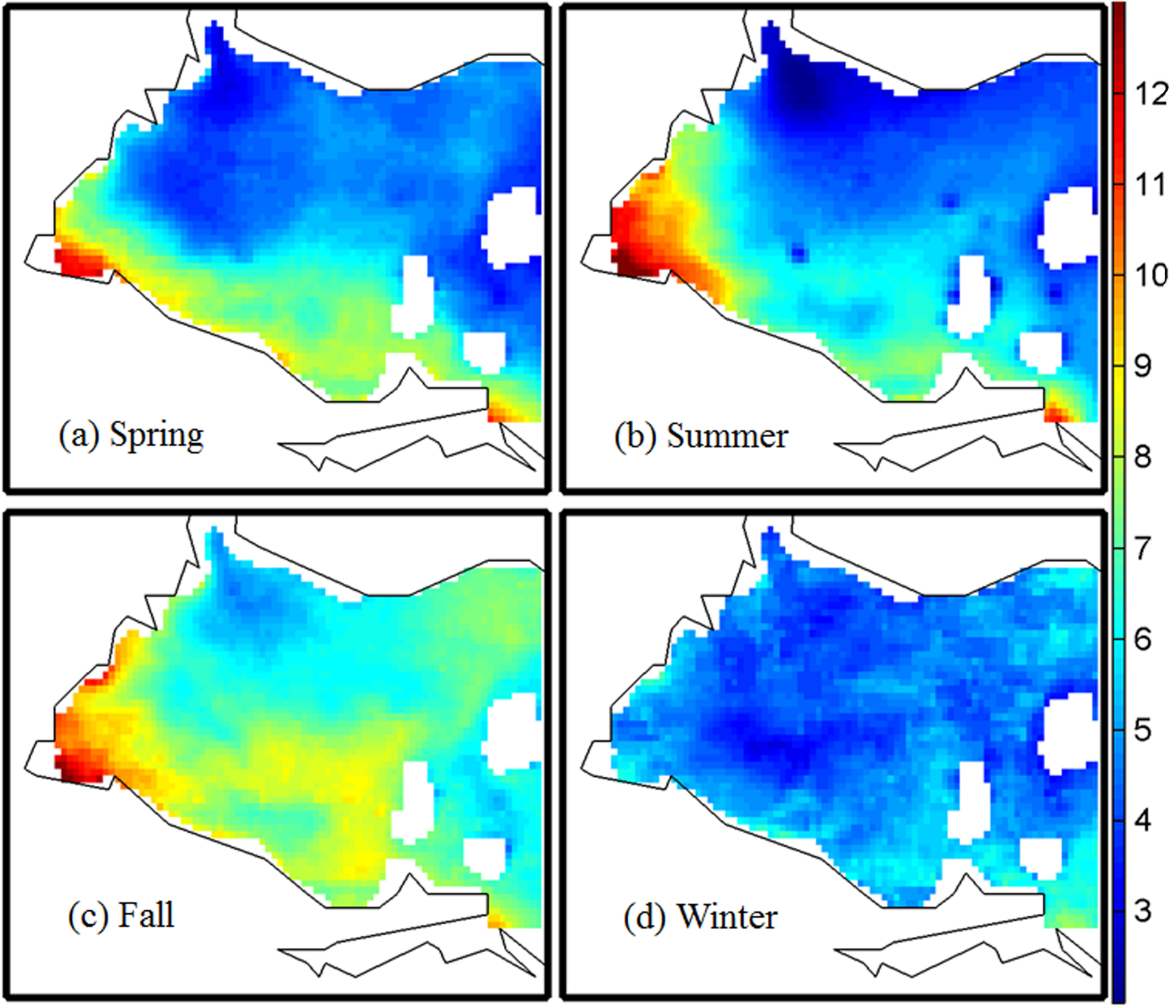
**Chl-a concentration (mg/L) in four seasons: (a) spring, (b) summer, (c) fall, and (d) winter.** Data were not available in white areas.

Fig 1 shows estimates of Chl-a concentrations over the western Lake Erie for the four seasons. Significant spatial and seasonal heterogeneities in both Chl-a were observed. For the Chl-a, the concentrations were higher in the south near shore area than in the north near shore area, with the highest level near the outlet of the Maumee River, followed by the Sandusky area. The concentrations were higher in spring, summer, and fall than in winter. In particular, summer during which high concentrations of Chl-a accumulated more in the Maumee River outlet area, while in the fall they span much wider area on the west and south side of the lake. These findings are consistent with Wayne and Stump [46], who observed similar spatial patterns of 13-year average Cyanobacterial Index concentration in western Lake Erie during summer and fall seasons. It is worthwhile to note that high concentrations of Chl-a were found near the outlet area of the Maumee River and Sandusky Bay and the south near shore area had a higher concentration than the north. This was likely due to the high nutrient loadings from the Maumee and Sandusky Rivers and the long residence time induced by low circulation in the western and southern basin, consistent with findings by Michalak et al. [25]. It was expected that winter would have lower concentrations of Chl-a than the other three seasons likely due to the low temperature and ice over western Lake Erie. The high concentrations of Chl-a in spring and fall were likely caused by warm and wet meteorological conditions and excessive fertilizers that washed off from agricultural land into the rivers by strong rainfall, as also indicated by Michalak et al. [25].

### Temporal variability of Chl-a

**Fig 2:**
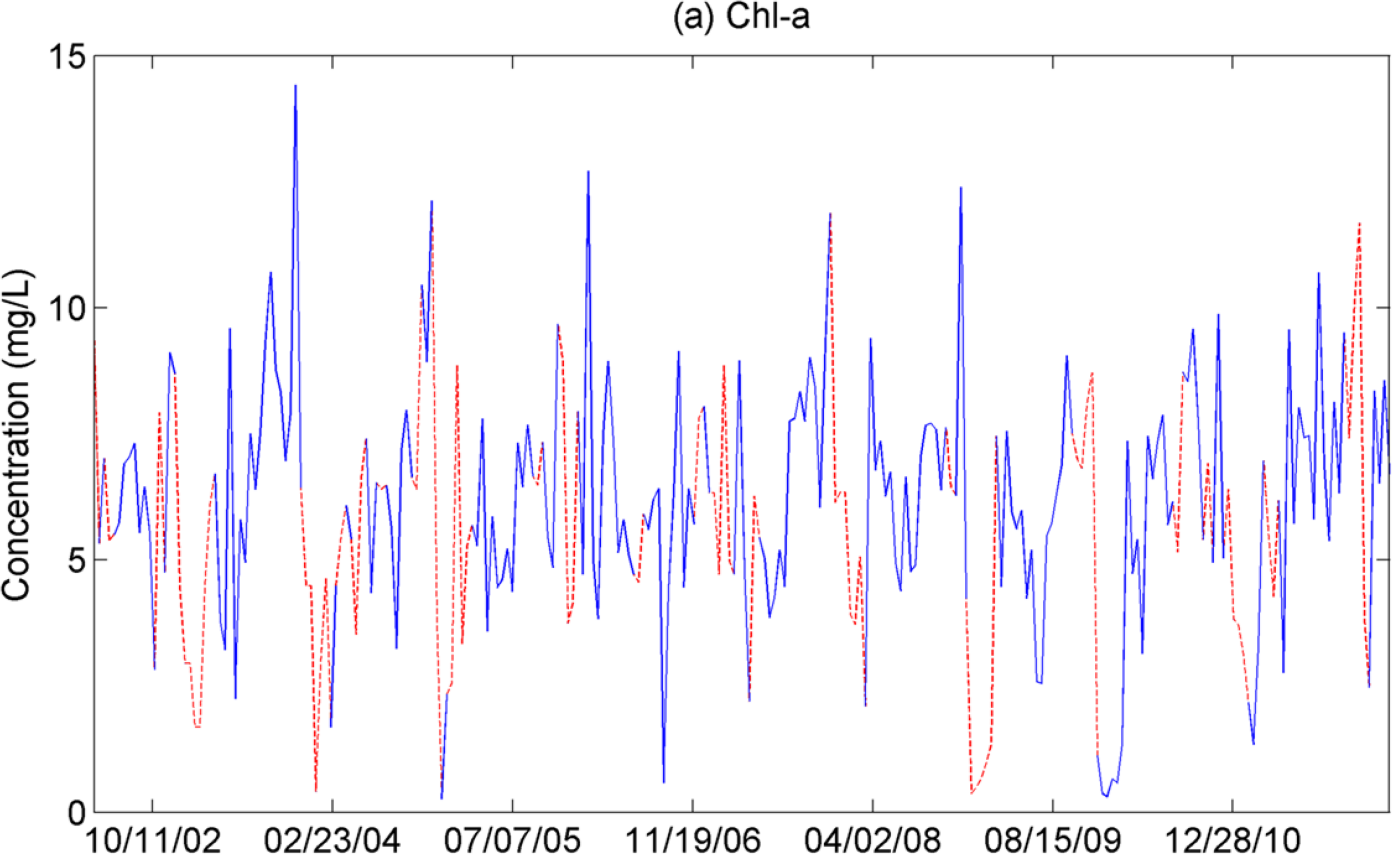
Spatial averaged Chl-a concentrations over western Lake Erie. The Chl-a series is from 5 January 2002 to 21 March 2012. Red lines are filled gaps with a mean of the available data within the ±14-day window of its neighbor years.

**Fig 3:**
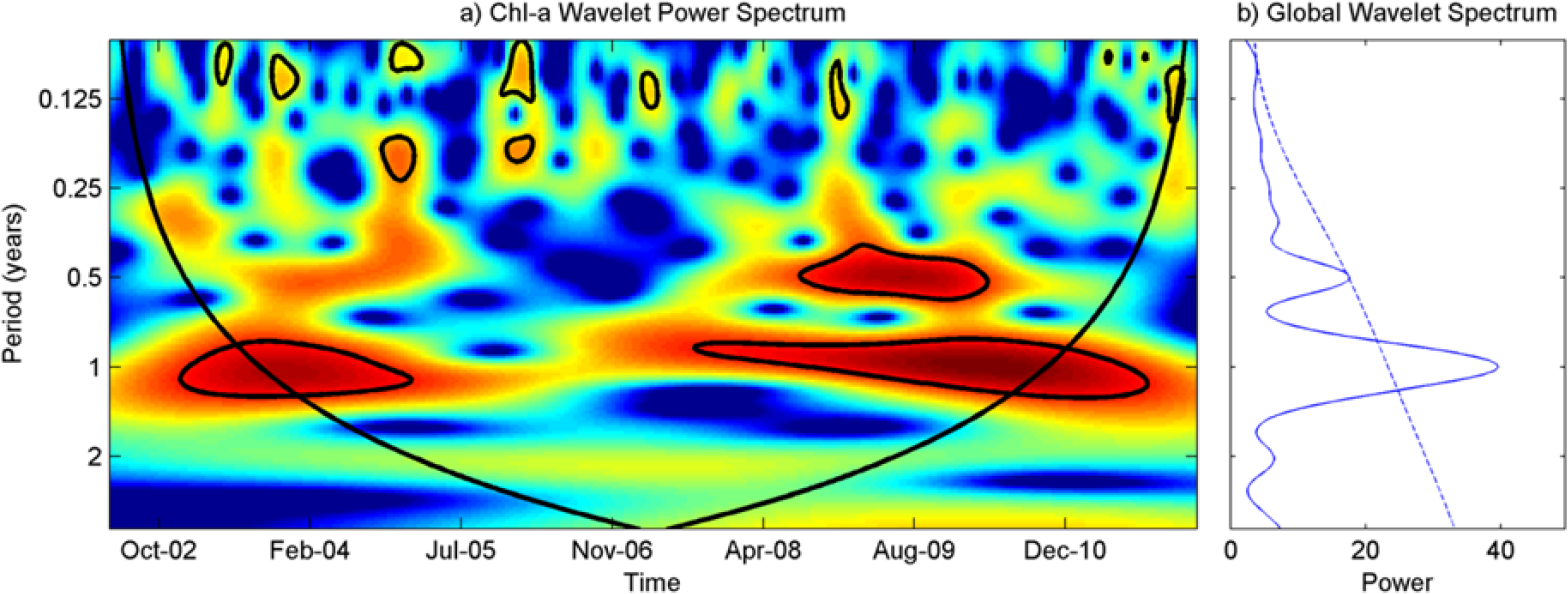
**Wavelet power spectrum of the spatial average Chl-a concentration: (a) wavelet power spectrum of Chl-a, (b) global wavelet spectrum of Chl-a.** The Chl-a series is from 5 January 2002 to 21 March 2012. The 5% significance level against red noise is shown as a thick contour. Outside the black cone contour is the region of the cone of influence (COI)–which is due to the finite length of the time series where the edge effects become important and distort the results.

Fig 2 shows time series plots of spatial averages of Chl-a over western Lake Erie. The global wavelet power spectrums revealed that the pattern of Chl-a was dominated by 0.5- and 1-year periods (Fig 3). The wavelet power spectra of Chl-a were significantly high from the year 2002 to 2005 and from 2007 to 2010 for a 1-year period and from 2008 to 2010 for a 0.5-year period. A strong power spectrum for a half year period in the Chl-a time series agreed with the finding of the high concentrations of Chl-a in spring and fall described the section above.

### Model estimates and variable importance

Environmental variables included in the analysis could be potentially correlated with each other. Using the VIF analysis described in the method section, the environmental variables with high multicollinearity were identified for each season and were excluded in the analysis when implementing MARS model (Table 2).

**Table 2:** Selected environmental variables for MARS model in each season based on the VIF analysis.

No	Spring	Summer	Fall	Winter
1	PCP	PCP	PCP	PCP
2	Wind	Wind	Wind	Wind
3	WTMP	WTMP	-	WTMP
4	Rs	Rs	Rs	Rs
5	Q	Q	Q	Q
6	TP	-	-	TP
7	SRP	SRP	SRP	SRP
8	NO23	NO23	NO23	NO23
9	TKN	TKN	TKN	TKN

**Fig 4:**
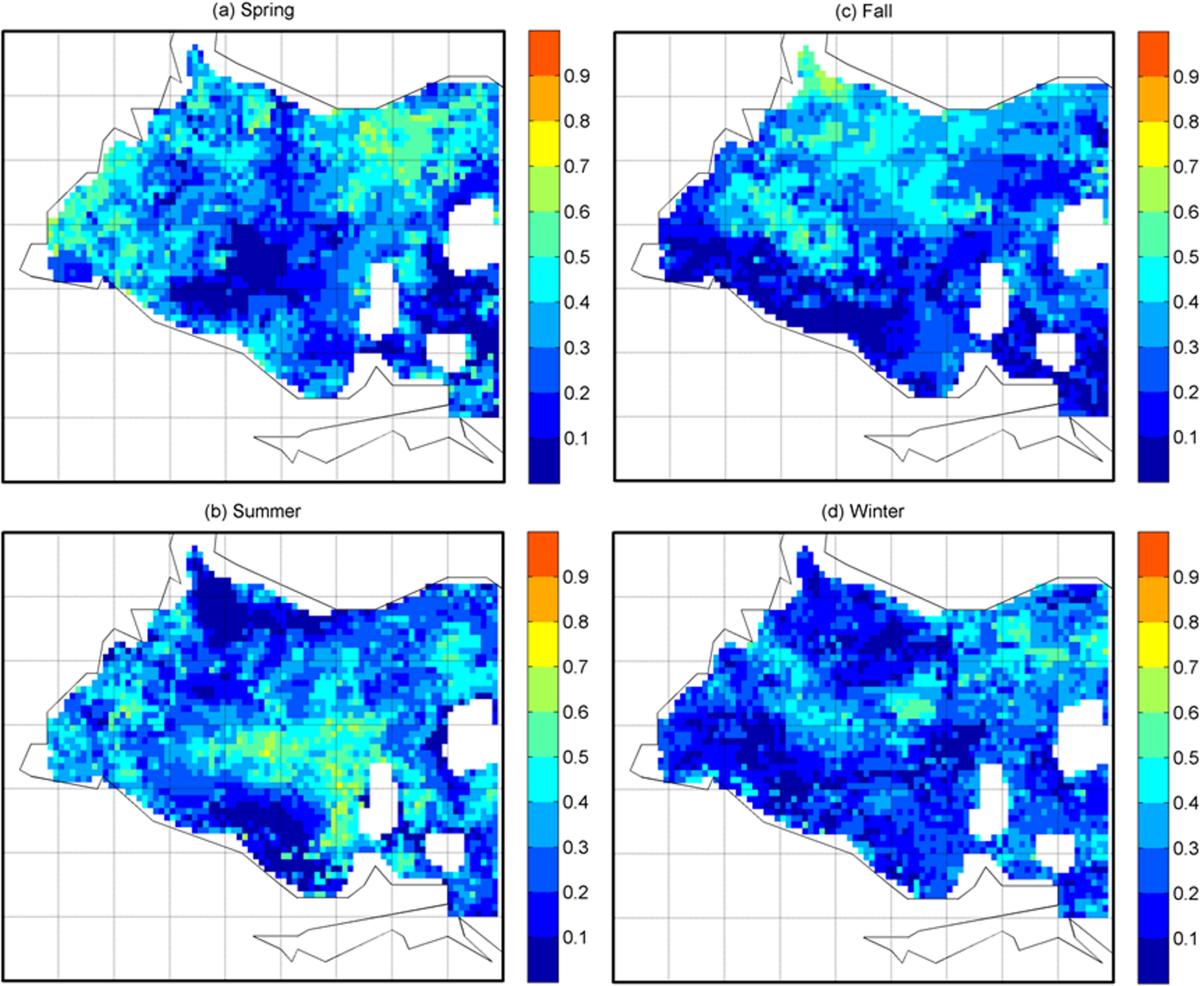
R^2^ values for MARS model to predict Chl-a for each pixel over western Lake Erie.

**Fig 5:**
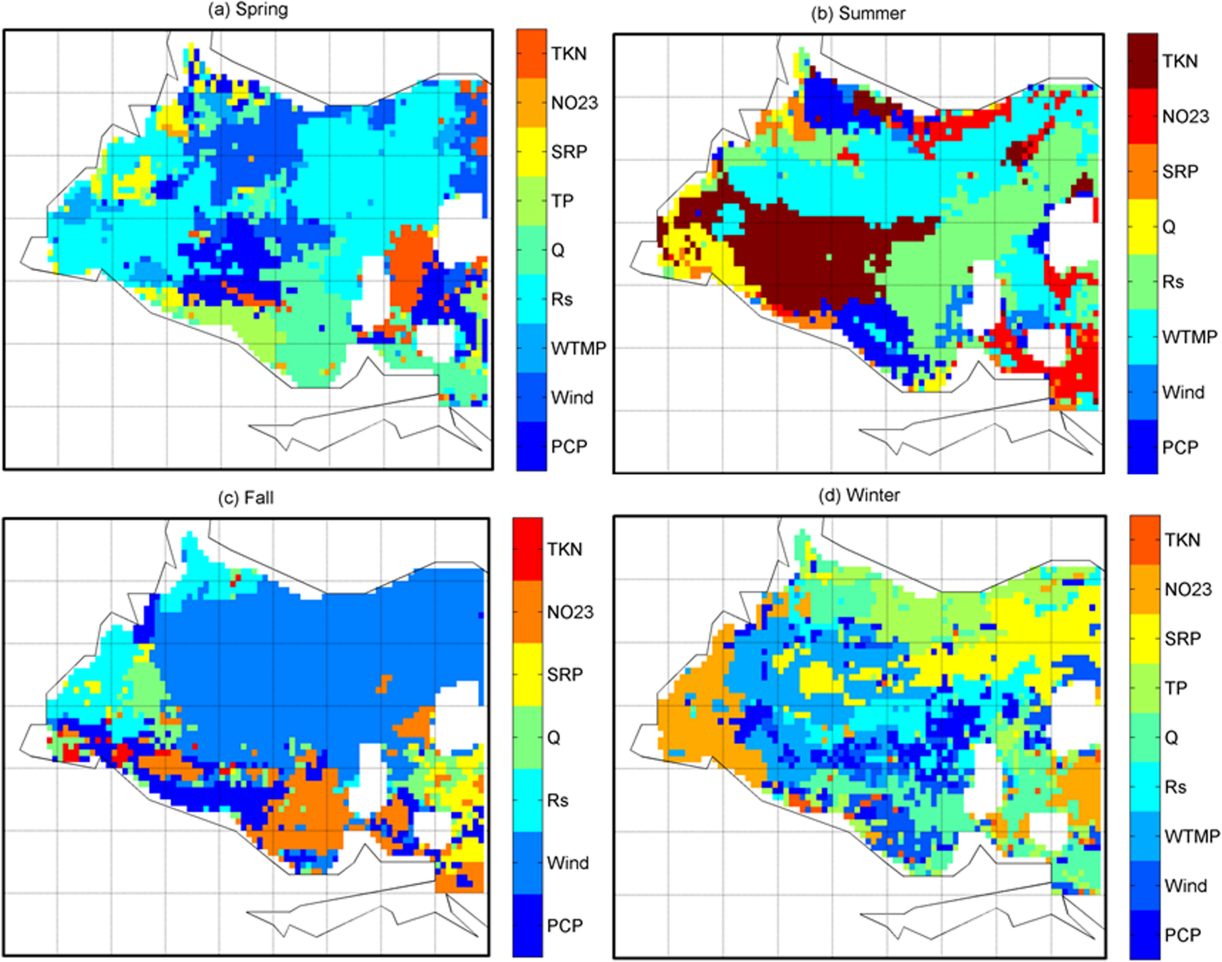
The environmental factor of the greatest importance for Chl-a identified by MARS at each pixel over western Lake Erie in four seasons: (a) spring, (b) summer, (c) fall, and (d) winter.

The selected environmental variables were used as predictors of Chl-a concentration in the MARS models at each pixel over the western Lake Erie. The MARS models’ explained variance to the total variance (R^2^) of the Chl-a concentration showed varying levels of performance in four different seasons over western Lake Erie (Fig 4). On average, the models of Chl-a explained about 29% of the variations in spring and summer, 26% of the variations in fall, and 24% of the variations in winter (Fig 4). The environmental variables of greatest importance for Chl-a were also identified by MARS for each season at the pixel scale over western Lake Erie (Fig 5). The variables of highest importance showed substantial heterogeneities across pixels in western Lake Erie. In fall, in south near shore and west area (Fig 5), where the highest Chl-a concentration was observed, the spatial distributions of the environmental variables of the highest importance showed substantial variations, implying that the mixed nutrient and meteorological factors (e.g. both ecological and phenological factors) may be related to observed HABs from the imagery. In spring, while the south area showed a high level of heterogeneity and complexity of environmental variables of importance, Rs was identified as the most important variable in most pixels in the west area. Whereas in summer, in the west area, where the highest level of Chl-a concentration was observed, Q was consistently found to be of high importance. In winter, Chl-a showed a very high degree of spatial heterogeneity. This was likely due to that in winter, the factors of associated with the extreme low Chl-a could be relatively random as a result of extremely low or even zero concentrations of Chl-a picked from satellite images. Table 3 summarizes the percentage of the variable of highest importance for Chl-a in four seasons over the western Lake Erie based on the MARS analysis. For Chl-a, the environmental variables of highest importance are mixtures of both ecological and phenological factors, wind and Rs in the spring, WTMP, Rs, and TKN in the summer, wind in the fall, and WTMP and Q in winter. It is worth noting that phenological factors, in particular, wind speed, were consistently found to be of high importance for Chl-a over large areas of western Lake Erie. This finding agreed with the expectation that a warm and quiescent environment with high light availability would create an ideal condition to develop HABs [47–49]; and also was consistent with the finding that the wind speed was an important factor associated with variability of cyanobacterial bloom [49] and hypoxia [30] in Lake Erie. While overall findings of the study agree with most previous studies on the importance of nutrient loadings as primary factors linking to blooms in Lake Erie [23, 25, 30], they also showed some inconsistencies with, for example, Stump et al.’s findings [23]. Such discrepancy was likely due to the addition of phenological factors (e.g. wind) in the analysis and the use of different analytical approaches. Our study suggests that phenological variables, such as wind, are likely important factors in the ecology and remote sensing based observations of Chl-a. High spatial heterogeneities in the distribution of Chl-a and relative importance of these factors across seasons were also noted, particularly in the in the west area of the lake in spring and Rs and Q were consistently found as the most important factors in these two seasons. However, we acknowledge the limitation of the finding as the nutrient loading information were from the river-based monitoring data, which are likely to impact results from places furthest from key rivers, although we think that results from key rivers and surrounding areas, which are more vulnerable to HABs, were less likely to be impacted by the limitation.

**Table 3:** Percentage of the environmental variable of greatest importance for Chl-a (%) in four seasons over all pixels in western Lake Erie. Values over 15% were highlighted with bold.

Variable	Chl-a			
	Spring	Summer	Fall	Winter
PCP	11.0	8.8	10.6	9.2
Wind	**16.7**	2.7	**54.7**	9.0
WTMP	4.7	**27.2**	-	**15.8**
Rs	**37.7**	**25.0**	9.1	9.0
Q	14.3	4.7	6.8	**15.0**
TP	4.8	-	-	13.0
SRP	4.3	3.7	4.7	13.8
NO23	0.7	9.3	13.0	13.9
TKN	5.7	**18.6**	1.1	1.4

Although the results offer important insights into relative importance of these environmental factors for remote-sensing based observations of HABs in western Lake Erie, some important caveats and limitations should be noted here. First, it is important to note that, although ‘varying’ importance of the two types of factors (ecological and meterological, or phenological) in relation to Chl-a across different seasons was reported in the present study, these factors play very different roles. Factors related to nutrient loading, are ecological in nature and provide driving forces for the growth and development of algae, whereas factors such as wind, are phenological and provide ‘external’ forces moving algae around. Both type of factors influence the remote sensing-based on Chl-a or HABs observations, but the natures of their roles are totally different. Second, there is a number of gaps in the Chl-a data derived from MERIS, while filled with KNN imputation algorithm, these data gaps may have caused a certain level, although likely minimum, of uncertainty in exploring the spatial-temporal variability of Chl-a. Third, unlike the spatially varying Chl-a data, the environmental factors are spatially constant. Those factors are either averaged over the entire western Lake Erie or over a few selected sites in this region, which might affect the predictive capacity of the MARS models, as indicated above. Despite these limitations, the study might provide some important insights into the dynamics of HABs in the west Lake Erie, particularly areas around key rivers, where important ecological data (e.g. nutrient loading) are monitored and used in the present study.

## Conclusion

This study examined spatial-temporal patterns of the Chl-a and the environmental factors associated with the HAB indicator in western Lake Erie. The Chl-a serie was dominated by the 0.5- and 1-year periods. At the pixel scale, Chl-a exhibited high heterogeneities in the spatial and temporal distribution and such heterogeneities were correlated with a mixture of both ecological and phenological factors of varying importance. In comparison with other studies, the consistency and differences of the findings of the study may suggest strengths and limitations of the remotely sensed imagery-based analysis, offering valuable information for future work.

## Supporting information

S1 File Chl-a estimates from the satellite image. The file contains the Chl-a estimate for the whole study area.
(MAT)

S2 File Environmental variables. The file contains environmental variables used in the analysis.
(CSV)

S3 File Time information for Chl-a estimates. This file contains time information for the Chl-a estimates.
(MAT)

S4 File Average Chl-a estimates. This file contains average Chl-a estimates across the study periods.
(MAT)

S5 File Spatial average Chl-a estimate. This file contains spatial average Chl-a estimates across the study periods.
(MAT)
